# Breast cancer-specific survival by molecular subtype in different age groups of women in Scotland

**DOI:** 10.1186/s13058-025-02012-x

**Published:** 2025-04-22

**Authors:** Ines Mesa-Eguiagaray, Sarah H. Wild, Linda J. Williams, Kai Jin, Sheila M. Bird, David H. Brewster, Peter S. Hall, Jonine D. Figueroa

**Affiliations:** 1 Usher Institute, College of Medicine and Veterinary Medicine, University of Edinburgh, Edinburgh, UK; 2 School of Public Health, Faculty of Medicine and Health, University of Sydney, Sydney, Australia; 3 Cambridge University’s MRC Biostatistics Unit, Cambridge, UK; 4 Cancer Research UK Edinburgh Centre, Institute of Genetics and Cancer, University of Edinburgh, Edinburgh, UK; 5 Division of Cancer Epidemiology and Genetics, National Cancer Institute, Bethesda, MD USA

**Keywords:** Breast cancer, Subtypes, Age, Prognosis

## Abstract

**Background:**

Age and molecular subtypes are important prognostic factors in breast cancer (BC). Here, we explore how age and molecular subtypes influence BC survival in Scotland.

**Methods:**

We analysed data from 71,784 women diagnosed with invasive BC in Scotland between 1997 and 2016, with follow-up until 31st December 2018 (median follow-up time = 5.5 years). Cox models estimated Hazard Ratios (HR) for BC-specific death by age group (with women of screening age, 50–69 years old, as the reference) within each molecular subtype, adjusting for prognostic factors. The cumulative incidence function was plotted to account for competing risks.

**Results:**

During the study period, 37% of women died, with 53% of deaths attributed to BC. Women aged 70 + years had increased BC-specific death compared to women aged 50 to 69 years with the same subtype. HRs (95% CI) were 1.49 (1.23–1.80) for luminal A, 1.39 (1.14 to 1.69) for luminal B tumours and 1.49 (1.15 to 1.94) for triple negative breast cancer (TNBC). Women aged < 50 years had lower risk of BC death in luminal A subtype only, with HR of 0.66 (0.51–0.86) compared to women aged 50 to 69 years. Competing risks analysis showed higher cumulative incidence of death from non-BC causes, particularly for women aged 70 + years with hormone positive subtypes. Stage, treatment, and molecular subtype were the strongest prognostic factors for BC-specific mortality across all ages.

**Conclusions:**

Age influences BC-specific mortality particularly within luminal subtypes. In contrast, other tumour characteristics and treatment are key prognostic factors for non-luminal subtypes. Future studies should investigate other markers of BC mortality particularly among over 70-year-olds, who account for 60% of BC deaths in the UK.

**Supplementary Information:**

The online version contains supplementary material available at 10.1186/s13058-025-02012-x.

## Background

Breast cancer (BC) is the leading cause of cancer mortality among women in over 100 countries and is currently the most common cancer globally, having overtaken lung cancer [1]. Age is an important risk factor for BC, with nearly half of cases in the United Kingdom (UK) diagnosed in women aged 50 to 70 years and a third of cases in women over 70 years of age [2]. Further, 90% of BC deaths in the UK occur in women over 50 years at the time of death, with 60% of these deaths in women over 70 years of age [2]. Given the increasing life expectancy and projections that by the middle of the 21st century almost a third of the UK population will be over 70 years of age [3], this age group is an increasingly important demographic for public health.

Despite the role of age in BC incidence and prognosis, chronological age alone should not be used as a reason to withhold specific treatments [4] since the benefit from chemotherapy is independent of age [5]. Further, age is not the only important prognostic factor in BC, multiple previous studies have also highlighted the role of the BC molecular subtypes [6], with luminal subtypes having more favourable prognosis than triple negative BC (TNBC) or human epidermal growth factor receptor (HER2) enriched subtypes [7–12]. However, fewer studies have investigated how age influences BC prognosis for the different molecular subtypes, and those who had lacked adjustment for important confounding factors, such as socio-economic status, comorbidities and competing risks.

The Scottish cancer registry is an excellent resource to address this gap, as it includes long-term data on oestrogen receptor (ER) status (since 1997) and progesterone receptor (PR) and HER2 status (since 2009)- almost a decade earlier than other UK national registries [6]. Further, the cancer registry data can be linked to other health electronic records to enable adjustment for important confounders. We have previously shown that age is an important predictor of incidence of BC tumour subtype in Scotland [6]. Here, we aimed to investigate the role of age on BC survival across the different molecular subtypes. Specifically, we assessed (1) how survival outcomes vary by molecular subtypes across different age groups, (2) whether age modifies the prognostic impact of the molecular subtypes and (3) the association of other tumour characteristics, screening, treatment, socio-economic status, comorbidities and competing risks on survival.

## Methods

### Data and cohort definition

In this retrospective cohort study, women aged 20 years or older diagnosed with a first primary invasive BC (defined using the International Classification of Diseases, 10th revision code [ICD10] of C50) between 1997 and 2016 in Scotland were identified from the Scottish cancer registry data [13]. The Scottish Cancer registry was established in 1958, and electronic data was available from 1981. The registry covers all Scottish residents registered with a general practitioner (GP) reason why the coverage of the registry is really high, estimated at 98% for BC cases independently of age [14]. Women with missing postcodes, living outside Scotland, or who were older than 99 years (*n* = 55), with unknown vital status (*n* = 154), with BC diagnosed by death certificate only (*n* = 125) or who had the same date of incidence as date of death (*n* = 99) were excluded (0.6% of the total sample). The final study population consisted of 71,784 women.

For each woman, information on year of BC diagnosis (in 5- year calendar groups), age at BC diagnosis, Scottish region defined by Scottish geographical regions (North, South East and West), tumour characteristics (stage, tumour grade, tumour size, nodal status, method of detection, ER, PR and HER2 status) and binary categories of treatment (surgery, radiotherapy, chemotherapy and hormone therapy) was ascertained by the cancer registry from medical and pathology records. Information on targeted treatment for HER2 tumours was not available.

An area-based measure of socio-economic status at diagnosis, **the Scottish Index of Multiple Deprivation (SIMD)**, was derived for all women within the cancer registry with a Scottish postcode. The SIMD deprivation measure is based on 7 domains: income, employment, health, education, crime, access to services and housing [15]. SIMD is often expressed in quintiles and we used SIMD quintile to compare women in the 20% most deprived areas (quintile 1) with women in the 20% least deprived areas (quintile 5). The **Charlson index of comorbidity**, a measure that consists on a weighted index that takes into account the number of comorbidities and the severity of each comorbidity [16], was linked to the registry data from hospital inpatient records.

ER and PR status are measured by immunohistochemistry (IHC). HER2 status was assessed using a combination of IHC with fluorescent in-situ hybridisation for equivocal (2+) cases and the three markers were defined as positive, negative or unknown. **BC subtypes** were derived from the combination of ER, PR and HER2 based on the definition adopted in the St. Gallen 2011 consensus [12] and served as surrogates for the four intrinsic molecular subtypes of BC. As the registry lacks information for Ki67, tumour grade was used as a marker for cell proliferation. ER + and/or PR + and HER2- (grade 1 and 2) tumours were defined as luminal A, ER + and/or PR + and HER2- (grade 3) and ER + and/or PR + and HER2 + as luminal B, ER- and PR- and HER2 + as HER2-enriched and ER- and PR- and HER2- as TNBC.

**Age at diagnosis** was stratified in three categories based on the age at which women are routinely invited for mammography screening in Scotland: women aged less than 50 years, women aged 50 to 69 years (current age range for routine screening) and women aged 70 years or older (who only receive screening on request). From 1997 to 2004 the age range for routine breast screening was 50–64 years. **Tumour grade** was defined as low grade or well differentiated (grade I), medium grade or moderately well differentiated (grade II) and high grade or poorly differentiated (grade III). Information on **method of detection** was used to categorise each diagnosed tumour as screen-detected or not screen-detected (which included all remaining options: clinical examination, incidental finding and unknown).

**Stage** was derived from individual tumour (T), nodes (N) and metastasis (M) clinical and pathological stages. Clinical TNM stage was available during the entire study period but pathological TNM stage was only available from 2005. As a general principle, pathological TNM stage was prioritised over clinical stage as it tends to be more accurate. There were some exceptions: (1) for M stage except when clinical M stage was unknown or pathological M stage indicated metastasis (stage IV) and clinical stage did not, (2) if the woman had neoadjuvant therapy [radiotherapy, chemotherapy or hormone therapy (HT)] at least 4 weeks before surgery and (3) if clinical T stage was T4 indicating primary tumour involvement of chest wall or skin which is often obvious at clinical examination. Following the rules above and using pathological tumour size and the number of positive nodes to complete missing pathological T and N variables a final TNM stage variable was derived and categorised as I, II, III and IV following the American Joint Committee on Cancer (AJCC) 8th Edition Cancer Staging manual [17]. 

### Statistical methods

**Breast cancer specific survival (BCSS)** was the primary outcome of the analysis. Primary and secondary causes of death are derived from death records linked to the cancer registry [18]. ICD9 174 and ICD10 C50 codes from primary cause of death were used to derive BC specific death using the approach described by Skyrud et al. [18]. Other primary causes of death were regarded as censored observations for the calculation of BCSS. Duration of follow-up was defined as time from date of diagnosis of BC to the earliest date of death, 31st December 2018 for women still alive at the end of the study period or embarkation date if women moved from Scotland.

Non- parametric **Kaplan-Meier (KM)** estimates [19] were used to estimate BCSS at 5 years by ER status and for each IHC defined molecular subtype for all women and by age group, addressing the question of how age and tumour subtype influences BCSS. **Cox proportional hazards models** [20] were fitted to investigate the association between the molecular subtypes and age category (as main predictors) and BC-specific mortality over time after controlling for other covariates and to assess which factors were more important for prognosis. **Preliminary models** included the two main exposures (ER status or the IHC defined molecular subtypes and age category at diagnosis), year of diagnosis and Scottish NHS region (model 1). Model 2 included model 1 plus tumour characteristics. Model 3 included model 2 plus treatment regimens and model 4 included model 3 plus deprivation and comorbidity measures. We also tested for an interaction between ER status and age category and between IHC defined subtypes and age category by comparing models with and without the interaction. The models were restricted to those women with information for all covariates (*n* = 51,140, 71% of the total). **Sensitivity analyses** was also conducted to estimate interactions between the model covariates with time and are presented (for the ER models only). Time varying covariates were used when the PH assumption from visual Schoenfeld residuals did not hold (this assumption is often violated in the presence of markers for BC) [21, 22].

Given that a significant interaction between molecular subtypes (either defined using ER status or the St. Gallen’s criteria) and age was found and that the PH assumption did not hold for the molecular subtypes, the **main models** presented were stratified by molecular subtypes to assess the effect of **age** (unadjusted and adjusted for other covariates) on BC-specific death within each molecular subtype. Hazard Ratio (HR) with 95% CI are presented for all models.

Competing risks analysis was performed to investigate whether the risk of early death from other causes differed between the molecular subtypes and age groups by plotting the **cumulative incidence function** [23] stratified by age and the molecular subtypes, with deaths from causes other than BC as the competing risk. The cumulative incidence function partitioned the contributions to mortality from BC-specific death and other causes of death and was estimated using Fine and Gray subdistribution hazard function [24]. All tests were 2-sided and deemed significant at the 5% level. Analyses were performed in Stata version 15/IC [25].

## Results

### Characteristics of cohort

Of the 71,784 women diagnosed with BC between 1997 and 2016 in Scotland with a median follow-up of 5.5 years, 26,280 (37%) died during the study period (Table 1). Of those who died, 53% had BC as their underlying cause of death. Differences in survival were observed by age group. Over 75% of women aged less than 70 years were alive at the end of the follow-up whereas only 35% of women 70 years and older survived. Among women younger than 50 years of age at BC diagnosis who died, 86% died of BC. Proportions of other causes of death were 43% and 57% respectively among women aged 50 to 69 years and older than 70 years at BC diagnosis.

**Table 1:** Individual characteristics, tumour characteristics, treatment regimens and mortality figures for the population of women diagnosed with BC in Scotland (1997 to 2016) by age group

	< 50 years (*N* = 14,379) [20%]	50–69 years (*N* = 35,592) [50%]	70 years or older (*N* = 21,813) [30%]	Total (*N* = 71,784)
NHS Region of Scotland				
North	3,639 (25%)	9,288 (26%)	5,665 (26%)	18,592 (26%)
South East	4,020 (28%)	9,769 (27%)	6,044 (28%)	19,833 (28%)
West	6,720 (47%)	16,535 (47%)	10,104 (46%)	33,359 (46%)
Year of diagnosis				
1997–2001	3,324 (23%)	7,741 (22%)	5,239 (24%)	16,304 (23%)
2002–2006	3,543 (25%)	8,319 (23%)	5,427 (25%)	17,289 (24%)
2007–2011	3,739 (26%)	9,473 (27%)	5,467 (25%)	18,679 (26%)
2012–2016	3,773 (26%)	10,059 (28%)	5,680 (26%)	19,512 (27%)
SIMD quintile				
Least deprived	3,218 (23%)	7,570 (21%)	4,101 (19%)	14,889 (21%)
4	3,080 (21%)	7,594 (21%)	4,252 (20%)	14,926 (21%)
3	2,878 (20%)	7,387 (21%)	4,593 (21%)	14,858 (20%)
2	2,726 (19%)	6,803 (19%)	4,729 (21%)	14,258 (20%)
Most deprived	2,477 (17%)	6,238 (18%)	4,138 (19%)	12,853 (18%)
Charlson Index, mean (SD)	0.02 (0.20)	0.05 (0.26)	0.11 (0.39)	0.06 (0.30)
No. comorbidities∆				
0	14,157 (99%)	34,263 (96%)	19,770 (91%)	68,190 (95%)
1	164 (1%)	1,096 (3%)	1,691 (8%)	2,951 (4%)
2 or more	58 (0%)	233 (1%)	352 (1%)	643 (1%)
TNM stage				
I	3,851 (27%)	15,576 (44%)	4,682 (22%)	24,109 (34%)
II	6,111 (42%)	11,875 (33%)	7,256 (33%)	25,242 (35%)
III	2,573 (18%)	4,221 (12%)	3,540 (16%)	10,334 (14%)
IV	601 (4%)	1,398 (4%)	1,612 (7%)	3,611 (5%)
Unknown	1,243 (9%)	2,522 (7%)	4,723 (22%)	8,488 (12%)
Tumour grade				
Grade I	1,196 (8%)	5,625 (16%)	1,863 (8%)	8,684 (12%)
Grade II	5,012 (35%)	15,063 (42%)	7,902 (36%)	27,977 (39%)
Grade III	6,658 (46%)	11,237 (32%)	5,590 (26%)	23,485 (33%)
Unknown	1,513 (11%)	3,667 (10%)	6,458 (30%)	11,638 (16%)
Tumour size (in cm)				
Less than 10 mm	1,108 (8%)	5,543 (16%)	1,016 (5%)	7,667 (11%)
10 to 20 mm	5,034 (35%)	14,571 (41%)	4,698 (22%)	24,303 (34%)
More than 20 mm	6,164 (43%)	11,014 (31%)	6,404 (29%)	23,582 (33%)
Unknown	2,073 (14%)	4,464 (12%)	9,695 (44%)	16,232 (22%)
Positive nodal status?				
Yes	6,216 (43%)	10,858 (30%)	4,769 (22%)	21,843 (30%)
No	7,172 (50%)	21,674 (61%)	7,525 (34%)	36,371 (51%)
Unknown	991 (7%)	3,060 (9%)	9,519 (44%)	13,570 (19%)
Diagnosed through screening?				
Yes	269 (2%)	17,101 (48%)	2,143 (10%)	19,513 (27%)
No	13,786 (96%)	17,877 (50%)	19,138 (88%)	50,801 (71%)
Unknown	324 (2%)	614 (2%)	532 (2%)	1,470 (2%)
ER status				
Positive	10,505 (73%)	28,385 (80%)	16,113 (74%)	55,003 (77%)
Negative	3,188 (22%)	5,655 (16%)	2,855 (13%)	11,698 (16%)
Unknown	686 (5%)	1,552 (4%)	2,845 (13%)	5,083 (7%)
PR status*				
Positive	3,096 (51%)	8,471 (53%)	4,499 (50%)	16,066 (51%)
Negative	1,482 (25%)	3,482 (22%)	1,868 (21%)	6,832 (22%)
Unknown	1,458 (24%)	3,997 (25%)	2,612 (29%)	8,067 (26%)
HER2 status*				
Positive	1,142 (19%)	2,082 (13%)	981 (11%)	4,205 (14%)
Negative	4,466 (74%)	12,337 (77%)	6,571 (73%)	23,374 (75%)
Unknown	428 (7%)	1,531 (10%)	1,427 (16%)	3,386 (11%)
Molecular subtype*				
Luminal A	2,337 (39%)	8,464 (53%)	4,623 (51%)	15,424 (50%)
Luminal B	2,147 (36%)	3,888 (24%)	1,893 (21%)	7,928 (26%)
HER2-enriched	310 (5%)	654 (4%)	321 (4%)	1,285 (4%)
TNBC	802 (13%)	1,388 (9%)	705 (8%)	2,895 (9%)
Unknown	440 (7%)	1,556 (10%)	1,437 (16%)	3,433 (11%)
Surgery				
Yes	13,753 (96%)	33,509 (94%)	13,099 (60%)	60,361 (84%)
No	527 (4%)	1917 (5%)	8,389 (38%)	10,833 (15%)
Unknown	99 (< 1%)	166 (< 1%)	325 (2%)	590 (< 1%)
Radiotherapy				
Yes	9,906 (69%)	24,164 (68%)	7,674 (35%)	41,744 (58%)
No	3,738 (26%)	9,842 (28%)	13,221 (61%)	26,801 (37%)
Unknown	735 (5%)	1,586 (4%)	918 (4%)	3,239 (5%)
Chemotherapy				
Yes	10,539 (73%)	14,180 (40%)	1,757 (8%)	26,476 (37%)
No	3,574 (25%)	20,622 (58%)	19,332 (89%)	43,528 (61%)
Unknown	266 (2%)	790 (2%)	724 (3%)	1,780 (2%)
Hormone therapy				
Yes	9,298 (65%)	25,953 (73%)	16,671 (76%)	51,922 (72%)
No	4,065 (28%)	7,583 (21%)	3,971 (18%)	15,619 (22%)
Unknown	1,016 (7%)	2,056 (6%)	1,171 (5%)	4,243 (6%)
Neoadjuvant therapy				
Yes	11,562 (80%)	31,948 (90%)	20,025 (92%)	63,535 (88%)
No	2,817 (20%)	3,644 (10%)	1,788 (8%)	8,249 (12%)
Vital status at the end of follow-up				
Dead	3,213 (22%)	8,894 (25%)	14,173 (65%)	26,280 (37%)
Alive	11,166 (78%)	26,698 (75%)	7,640 (35%)	45,504 (63%)
Breast cancer death^				
Yes	2,748 (86%)	5,095 (57%)	6,139 (43%)	13,982 (53%)
No	465 (14%)	3,799 (43%)	8,034 (57%)	12,298 (47%)

*restricted to years 2009 to 2016 (total *n* = 30,965, by age group: <50 years (*N* = 6,036), 50–69 years (*N* = 15,950) and 70 years or older (*N* = 8,979). ∆ Number of comorbidities prior to BC diagnosis. ^Reported for patients who died during the follow-up period (*n* = 26,280). Parenthesis () are column percentages and brackets [] are row percentages. ER = oestrogen receptor, HER2 = human epidermal growth factor 2, NHS = National Health Service, PR = progesterone receptor, SD = standard deviation, SIMD = Scottish Index of Multiple Deprivation, TNBC = triple negative breast cancer, TNM = tumour, nodes, metastases

Over 75% of tumours in the study population were ER+ (*N* = 55,003) with half of these tumours diagnosed in women of screening age 50 to 69 years (*N* = 28,385). Younger women (< 50 years) and older women (> 70 years) were slightly less likely to be diagnosed with an ER + tumour but the vast majority of tumours were hormone positive in all age groups (73% and 74% respectively compared to 80% for 50–69-year-old women) (Table 1). The distribution of molecular subtypes differed between age groups with women younger than 50 years having higher proportions of luminal B and TNBC tumours (36% and 13% respectively) and a smaller proportion of luminal A tumours (39%) than women aged 50 years or older. The proportion of HER2-enriched tumours was similar across age groups, accounting for approximately 4% of all tumours.

Other tumour characteristics also differed by age group. Women of screening age (50 to 69 years) had almost half of their tumours diagnosed through screening and had characteristics associated with less aggressive disease, including lower tumour stage, grade and size and lower positive nodal status when compared to women younger than 50 years. Women aged 70 years or older were more likely to have missing tumour characteristics than women younger than 70 years.

Treatment differences were also observed between age groups (Table 1). Women aged 70 years or older were less likely to have surgery, radiation and chemotherapy and more likely to have hormone therapy and neoadjuvant hormone therapy than women younger than 70 years.

### Breast cancer-specific survival by molecular subtypes across different age groups

BCSS was highest amongst women aged 50 to 69 years, particularly for those diagnosed with ER + tumours (92.1% survival at 5 years), and lowest amongst women aged 70 years or older diagnosed with ER- tumours (55.4% survival at 5 years) (Table 2). Women younger than 50 years had only slightly lower BCSS compared to those aged 50–69, while women aged 70 years or older had lower survival across all tumour subtypes, particularly for HER2-enriched and TNBC tumours. Among all IHC-defined subtypes, TNBC had the lowest survival (72.6% survival at 5 years), regardless of age.

**Table 2:** Breast cancer specific survival estimates (in %) at 5 years after diagnosis (with 95% CI) by ER status and IHC defined subtypes for women of all ages (total) diagnosed in Scotland and by age group

Breast cancer specific survival	< 50 Years	50–69 Years	70 Years or older	Total
ER+				
deaths/cases	1,689/10,505	3,195/28,385	3,656/16,113	8,540/55,003
5-years % (95% CI)	89.2 (88.6, 89.9)	92.1 (91.7, 92.4)	77.1 (76.4, 77.9)	87.4 (87.1, 87.7)
ER-				
deaths/cases	845/3,188	1,421/5,655	1,153/2,855	3,419/11,698
5-years % (95% CI)	75.1 (73.5, 76.7)	75.1 (73.9, 76.4)	55.4 (53.4, 57.5)	70.6 (69.7, 71.5)
Luminal A				
deaths/cases	113/2,337	274/8,464	576/4,623	963/15,424
5-years % (95% CI)	93.6 (92.1, 94.9)	95.5 (94.8, 96.0)	81.6 (80.0, 83.1)	91.3 (90.1, 91.9)
Luminal B				
deaths/cases	228/2,147	319/3,888	367/1,893	914/7,928
5-years % (95% CI)	86.2 (84.2, 88.0)	88.8 (87.5, 90.1)	73.3 (70.5, 75.8)	84.5 (83.5, 85.5)
HER2-enriched				
deaths/cases	39/310	85/654	102/321	226/1,285
5-years % (95% CI)	81.5 (74.9, 86.6)	81.7 (77.4, 85.3)	58.4 (51.1, 65.0)	76.0 (72.7, 78.8)
TNBC				
deaths/cases	151/802	219/1,388	219/705	589/2,895
5-years % (95% CI)	74.5 (70.5, 78.1)	78.6 (75.7, 81.1)	57.4 (52.4, 62.1)	72.6 (70.5, 74.6)

Figures presented are 5-year KM survival probabilities with 95% CI calculated using estimated standard error (SE). CI = confidence interval, ER = oestrogen receptor, HER2 = human epidermal growth factor 2, TNBC = triple negative breast cancer

Data from the preliminary fully adjusted models (Supplementary Tables 1 and 2) showed that age and molecular subtypes were independent significant prognostic factors for BC-specific death and supported an interaction between age and molecular subtype. Table 3 presents the effect on BCSS of each age group (compared to the reference 50–69 years old group) stratified by molecular subtype. After adjusting for covariates, women younger than 50 years were less likely to die from BC-specific death compared to women aged 50 to 69 years, particularly for ER + tumours [HR = 0.89 (95%CI: 0.82 to 0.96)], luminal A tumours [HR = 0.66 (95%CI: 0.51 to 0.86)] and HER2-enriched tumours, although the latter association with age was not statistically significant.

**Table 3:** Unadjusted and adjusted Cox models for breast cancer specific mortality stratified by molecular subtype showing the effect of each age group (compared to the reference group of women aged 50 to 69 years) in each molecular subtype

Subtype		Unadjusted HR (95% CI) *P* value		Adjusted HR (95% CI) *P* value	
	Deaths/cases*	< 50 years	70 years or older	< 50 years	70 years or older
**ER+**	**5,238/42,146**	**1.43 (1.33–1.53)**	**2.35 (2.21–2.50)**	**0.89 (0.82–0.96)**	**1.34 (1.24–1.45)**
**ER-**	**2**,**378/9**,**105**	1.07 (0.96–1.18)	**2.09 (1.90–2.29)**	0.92 (0.83–1.02)	**1.30 (1.16–1.46)**
**Luminal A**	**723/13**,**755**	**1.42 (1.11–1.81)**	**4.60 (3.91–5.42)**	**0.66 (0.51–0.86)**	**1.49 (1.23–1.80)**
**Luminal B**	**753/7**,**132**	**1.21 (1.01–1.46)**	**2.62 (2.22–3.09)**	1.10 (0.90–1.34)	**1.39 (1.14–1.69)**
**HER2-expressed**	**147/1**,**034**	0.88 (0.55–1.42)	**2.86 (2.01–4.09)**	0.64 (0.39–1.06)	0.72 (0.45–1.16)
**TNBC**	**453/2**,**519**	1.24 (0.98–1.56)	**2.32 (1.87–2.88)**	1.07 (0.84–1.37)	**1.49 (1.15–1.94)**

Footnote: *Number of deaths/cases restricted to the complete case analysis models (patients with all variables in the adjusted model). Adjusted model includes age at diagnosis, incidence year, NHS region, grade (only in the HER2-enriched and TNBC models), TNM stage, method of detection, surgery, radiotherapy, chemotherapy, hormone therapy (only for luminal models), SIMD and Charlson score index. HRs reported are for the comparison of each specific age group with the reference age group of 50 to 69 years. HRs in bold were statistically significant at the 0.1% level (*p* < 0.001). CI = confidence interval, ER=oestrogen receptor, HER2= human epidermal growth factor 2, HR = hazard ratio, NHS = National Health Service, SIMD = Scottish Index of Multiple Deprivation, TNBC = triple negative breast cancer, TNM = tumour, nodes,metastases

For women aged 70 years or older, the risk of BC-specific death was higher for all tumour subtypes compared to the 50 to 69 age group, for both ER + and ER- tumours (34% and 30% increased risk, respectively). This elevated risk remained after adjusting for all covariates, except for HER-2 enriched tumours (Table 3). The highest relative risk was observed among women aged 70 + with luminal A and TNBC tumours (HR = 1.49 for both).

### Tumour characteristics, screening, treatment, socio-economic status and comorbidities in BCSS

Women with ER- tumours had worse survival outcomes compared to women with ER + tumours, while luminal B, HER2-enriched and TNBC subtypes were associated with significantly higher risks of BC death (adjusted HRs of 2.04, 1.95 and 3.93 respectively, compared to luminal A tumours) (Supplementary Tables 1 and 2).

Apart from molecular subtype, other tumour characteristics -specifically, TNM stage and grade- as well as method of detection were the strongest prognostic factors. Adjusting for these factors (model 2) attenuated the HR for age and molecular subtype. Treatments (model 3) were also significant prognostic factors for BC-specific death, and their adjustment further reduced the HR for age and molecular subtype. However, adjustment for socio-economic status and comorbidities (model 4) had limited additional effect on the associations between BC-specific death with age and molecular subtype. A directed acyclic graph (DAG) of the effect of the main covariates in BCSS is presented in Supplementary Fig. 1.

### Time-varying effects of prognostic factors

To assess whether key prognostic factors changed over time, we present the results for the Cox model analysis incorporating time-varying covariates (Supplementary Table 3) with ER status as the main predictor. We observed a statistically significant interaction between time and ER status [HR for time*ER = 0.83 (95% CI: 0.81 to 0.85)], indicating that the HR for the comparison of ER- and ER + tumours decreased over time from diagnosis. HR estimates at different time points after diagnosis (1, 3, 5 and 10 years) are presented in Supplementary Table 4, which may help inform clinical decisions for improving survival.

### Competing risks of BC death amongst the different age groups and by subtype

Apart from BC, cardiovascular diseases (CVD), other cancers, chronic obstructive pulmonary disease (COPD) and Alzheimer’s disease or dementia were the most common primary causes of death in Scottish women diagnosed with BC (Supplementary Table 5). There was a clear relationship between age and distribution of cause of death, with a higher proportion of women aged 70 years or older dying from causes other than BC than among women aged less than 70 years. For example, CVD accounted for 22% of all deaths in women aged 70 years or older compared to 10% of total deaths in women aged 50 to 69 years and 2% in women aged less than 50 years at BC diagnosis (Supplementary Table 5).

**Fig. 1:**
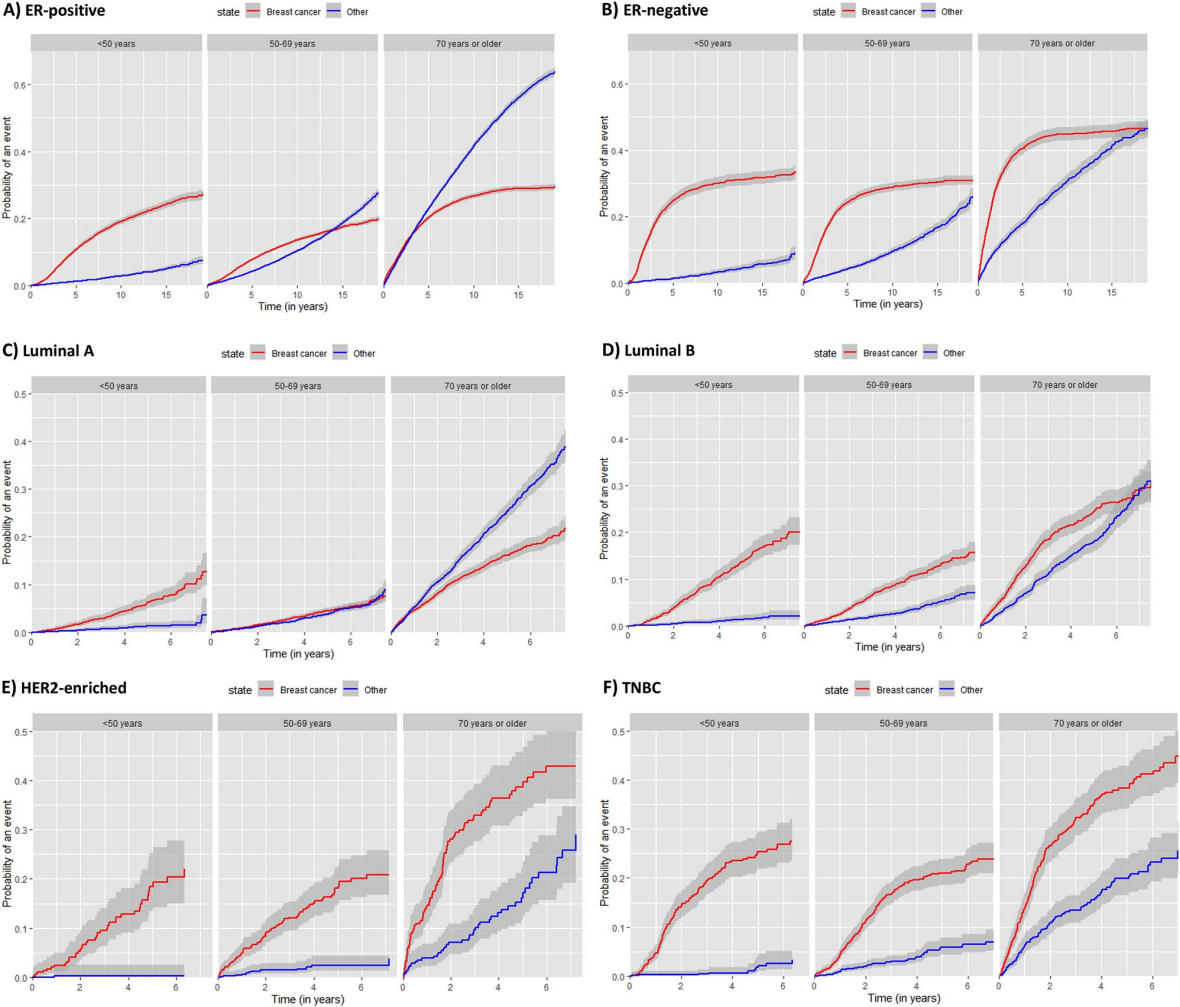
Cumulative incidence graph of BC death (breast: red line) and other cause of death (other: blue line) by age group for (**A**) ER + tumours (*n* = 55,003), (**B**) ER- tumours (*n* = 11,698), (**C**) luminal A tumours (*n* = 15,424), (**D**) luminal B tumours (*n* = 7,928), (**E**) HER2-enriched tumours (*n* = 1,285) and (**F**) TNBC tumours (*n* = 2,895)

The cumulative incidence function showed that the probability of dying from BC was highest in women aged 70 years or older with ER- tumours (Fig. 1b), particularly within the first 5 years after BC diagnosis. The probability of dying from BC for women with an ER- tumour aged less than 50 years was also slightly higher than for those aged 50 to 69 years with the same subtype (Fig. 1a). Competing risks of death (other than BC) were particularly high for the 70 years age group diagnosed with an ER + tumour for which the probability was similar to that of BC death in the first 5 years of follow-up and considerably higher after 5 years of follow-up. Younger women under 50 years had the lowest probability of dying from causes other than BC irrespective of ER status. Cumulative incidence functions by molecular subtypes show the highest probability of BC death amongst women aged 70 years or older with a TNBC or HER2-enriched subtype (Fig. 1e and f). Competing causes of death were more likely than BC death only for women aged 70 years or older diagnosed with a luminal A tumour (Fig. 1c), who contributed about 30% of all luminal A tumours. Women younger than 50 years had a slightly higher risk of BC death if diagnosed with a luminal tumour than women aged 50 to 69 years but were less likely to die from other causes.

## Discussion

In this large population-based study, including over 70,000 women diagnosed with BC in Scotland between 1997 and 2016- more than 51,000 of whom had complete data- we observed that BCSS survival and mortality differed by molecular subtype and age. Women with ER- tumours had nearly 20% lower BCSS than women with ER + tumours and a 44% higher risk of BC mortality, even after adjusting for other individual and tumour characteristics, treatments and comorbidities.

Our analysis of IHC-defined molecular subtypes showed that women with luminal A tumours had the highest five-year survival (91%), while women with TNBC subtypes had the worst BCSS (73% at five years). Additionally, we observed a 2-fold increased risk of BC-specific death for luminal B and HER2-enriched tumours and 4-fold increase for TNBC tumours when compared to luminal A tumours. These results are similar to those observed in the US [7, 26], Canada [27], Italy [28], Germany [8], Spain [29], Switzerland [30] and Norway [9]. However, direct international comparisons are challenging due to the differences in health care systems, breast screening programmes, access to targeted therapies and differences in the incidence of non-luminal subtypes.

### BCSS by molecular subtypes across different age groups

We observed differences in BCSS by age, with women aged 70 years or older having consistently higher risk of BC death compared to women of screening age (50 to 69 years), whereas women younger than 50 years had similar survival outcomes to those in the screening age group. Further, our data found evidence of survival outcomes differing by molecular subtype and age. A recent study from Norway of 21,384 women diagnosed from 2005 to 2015, showed younger age (< 40 years) was associated with higher breast cancer specific mortality among women with luminal A-like BC subtype, while old age was associated with increased death across all subtypes [9]. Notably, the 40 -to-49-year age group showed different associations with luminal A status, with a less aggressive disease characteristics than women < 40 years old (who had higher proportion of high grade HER2 + tumours). Our findings were consistent in showing women older than 70 years having worse BCSS for all tumour subtypes (except for HER2-enriched) compared to those 50–69 years. However, the magnitude of the age-related differences was smaller in Scotland than in Norway [9]. In contrast, our results showed that women younger than 50 years had lower risk of BC death compared to women of screening age among those with ER + tumours and luminal A tumours after adjusting for other covariates. Due to the limitations of our data and the small sample size for age groups < 40 and 40–49 years, we were unable to further stratify by these specific age groups. Future research should provide more granular association results by subtypes in women younger than 40 years old.

### Associations of tumour characteristics, screening, treatment, socio-economic status and comorbidities in BCSS

Tumour subtype, tumour characteristics (particularly stage and grade), screening and treatment attenuated the association of age with BCSS, showing that these make an important contribution to the association. In contrast, socio-economic status or comorbidities had little influence in BCSS. Women aged 70 years or older and women younger than 50 years had tumour characteristics of a more aggressive disease (higher proportions of late stage and higher-grade tumours) compared to women aged 50–69 years. Further, women aged 70 years or older were less likely to undergo surgery compared to women of 50–69 years of age. In general, the association of age category with BCSS was less strong than for subtype and tumour characteristics. Our data provide further evidence that tumour characteristics, screening and treatment are key factors in predicting BCSS.

Our analysis shows lower proportions of women aged 70 years and older treated with surgery, chemotherapy and radiotherapy than for women younger than 70 years. Although presence of comorbidities or frailty may influence treatment decisions, recent studies in the UK suggest that older women could benefit from more extensive use of chemotherapy and radiotherapy after primary surgical treatment in terms of survival and recurrence after diagnosis of BC [31]. However, almost half of women aged 70 + years diagnosed with BC who died did so from causes other than BC with CVD being a major contributor. Previous studies have indicated that some BC treatments increase CVD risk, such as radiotherapy to the left side of the chest and specific chemotherapy agents [32, 33]. Further research is needed to investigate risk of CVD among women who have received newer treatments to help identify the balance of risk and benefits of treatment for different populations of women with BC.

Older women are underrepresented in clinical trials [34]. Age is a key risk factor for BC incidence and there is an increasing number of studies investigating age-related biologic measures [35]. However, it is not clear which measures are the most important and whether their influence differs by tumour subtypes. Some studies have suggested frailty is an important factor in decisions about the appropriateness of chemotherapy [36] as a proxy for biological ageing [37] that could help identify those most likely to benefit from treatments. Interestingly, treatment with radiation or chemotherapy may accelerate ageing particularly among older patients [38, 39]. In our data, we observed missing stage and other tumor characteristics are likely to be higher in woman older than 70 which is likely due to these women being less likely to have surgery. Wyld et al. reported that ~ 20% of women older than 70 will have primary endocrine therapy in the UK [40]. This is likely due to clinicians having to manage frail or comorbid conditions in older women, which are more prevalent in this age demographic, where the morbidity risks of surgery are higher and life expectancy is likely to be relatively short [41]. Across different countries there is considerable variation in treatment strategies. However, at least across Europe little differences in survival were noted [42]. Although some trials have been aimed at improving treatment paradigms for older women [31] the evidence base is still limited for informed clinical decision making.

Data in Scottish women suggest that screening is an important indicator for BC prognosis regardless of molecular subtype [6, 43]. These results are in line with a recent study in Sweden reporting that women who participated in screening compared to those not participating in screening had a 41% reduction in the risk of BC death in the 10 years following diagnosis [44]. The potential for selection bias remains as women who accept invitations to screening are likely to differ from women that do not attend screening in ways that may influence survival.

### Strengths and limitations

The strengths of this study are the high quality of the Scottish cancer registry data [14] and linkage to mortality records that provides the opportunity to assess survival and mortality by BC subtype. As data on ER have been collected from 1997 and on PR and HER2 from 2009 this gives one of the longest follow-up periods of European cohorts. Further, possible confounders, such as method of detection, comorbidities and deprivation are not always recorded in other European and North American cancer registries and were included in the survival models and used to assess the effect of age in BCSS by subtype. Particularly, our study is further adjusted for socioeconomic factors and comorbidities using the SIMD and the Charlson score, although its inclusion only attenuated the association between age and BCSS for HER2 + tumours, suggesting that other factors contribute to the associations between age and BCSS or all-cause mortality. Other major strength of this study is the investigation of the proportional hazards assumption using time-varying models and the use of competing risks analyses.

A limitation of our study is the possibility of unmeasured confounders such as lifestyle factors (alcohol and tobacco consumption, physical activity), reproductive factors or anthropometric factors (such as body mass index). Further, there was no detailed treatment data available, particularly the registry lacked information on the type of surgery women had (mastectomy or lumpectomy), targeted treatment for HER2 + BCs which has likely affected survival of women with this tumour subtype in the last decades, nor data on BC recurrence. Additionally, we note that missingness of important confounders, which is particularly true for tumour characteristics in women older than 70 years -since these women are less likely to have surgery- might have an effect on the estimates. Another limitation is the use of subgroup analyses, which reduce statistical power and increase the risk of type I and type II errors.

Although IHC-defined subtypes are reasonably good proxies for the gold-standard of RNA-expression defined subtypes, the lack of mRNA expression assays for the classification of the molecular subtypes constitutes a clear limitation. Future research should aim to explain the differences observed in survival by mRNA expression molecular subtypes and age groups.

Finally, survival can be affected by lead time and length biases usually caused by the introduction of a national screening programme during the period of study [14]. These biases can explain survival improvements that are related to screening and not to changes in the number of deaths that are prevented or delayed. Most cancer registries do not record data about method of detection of a cancer, however the availability in the Scottish cancer registry allowed us to investigate the effect of screening in BCSS.

## Conclusion

Our study, using high quality population-based data from Scotland, highlights that age and molecular subtype are independent prognostic factors. Further, other tumour characteristics and treatments are also key prognostic factors for BCSS, regardless of age. Older women face a higher risk of breast cancer death even after adjusting for tumour characteristics, method of detection (screening), treatment, socio-economic factors and comorbidities. The increased mortality risk for women aged 70 years or older suggests that both biological factors and potential undertreatment may contribute to poorer outcomes. Additionally, competing causes of death, particularly cardiovascular diseases, were more prominent among older women (especially if they had a luminal A subtype) emphasizing the need for a comprehensive approach to patient management. These findings underscore the importance of age-specific strategies in breast cancer screening, treatment, and survivorship care to optimize outcomes for all patients. Scottish data have identified that many women with BC diagnosed over 70 years of age could potentially benefit from chemotherapy which require more systematic study [45]. In particular, it is not clear what measures of ageing, such as frailty, may inform who may or may not benefit from treatment. Future efforts need to further our understanding of factors that affect risk and benefits of treatments for different molecular subtypes to improve recommendations and outcomes (including patient reported outcomes) for the growing number of women with BC diagnosed over 70 years of age.

## Electronic supplementary material

Below is the link to the electronic supplementary material.

Supplementary Material 1

## Data Availability

The data that support the findings of this study are available through application to electronic Data Research and Innovation Service (eDRIS), a part of the Information Services Division of NHS Scotland. Restrictions apply to the availability of these data and any outputs presented here have been assessed and cleared for statistical disclosure by ISD Scotland. Outputs are however available from the authors upon reasonable request and with permission of ISD Scotland.
