# Supplementary material for: Breast cancer-specific survival by molecular subtype in different age groups of women in Scotland
Source: Breast Cancer Res. 2025 Apr 22;27:59. doi: 10.1186/s13058-025-02012-x (PMC12013176; doi:10.1186/s13058-025-02012-x)
Supplement: Supplementary file 1 — Supplementary Material 1 [file 13058_2025_2012_MOESM1_ESM.docx]

**Supplementary material**

Supplementary Table 1 Unadjusted and adjusted Cox models for the association of ER status with breast cancer death (page 1 of 2)

|  | **No women (%)** | **No deaths**  **(%)** | **Model 0**  **unadjusted** | **Model 1** | **Model 2** | **Model 3** | **Model 4** |
| --- | --- | --- | --- | --- | --- | --- | --- |
| **ER Status** |  |  |  |  |  |  |  |
| Positive | 42,146 (82%) | 5,238 (69%) | Ref | Ref | Ref | Ref | Ref |
| Negative | 8,994 (18%) | 2,354 (31%) | 2.31 (2.20-2.42) | 2.33 (2.22-2.45) | 1.66 (1.57-1.75) | 1.45 (1.34-1.56) | 1.44 (1.33-1.56) |
| **Age** |  |  |  |  |  |  |  |
| <50 years | 10,886 (21%) | 1,823 (24%) | 1.37 (1.30-1.45) | 1.27 (1.20-1.34) | 0.86 (0.81-0.91) | 0.89 (0.83-0.94) | 0.89 (0.84-0.95) |
| 50-69 years | 27,869 (55%) | 3,248 (43%) | Ref | Ref | Ref | Ref | Ref |
| 70 years or older | 12,385 (24%) | 2,521 (33%) | 2.23 (2.12-2.35) | 2.26 (2.14-2.38) | 1.59 (1.51-1.68,) | 1.36 (1.27-1.45) | 1.36 (1.27-1.45) |
| **NHS Scottish region** |  |  |  |  |  |  |  |
| West | 20,738 (41%) | 2,800 (37%) | Ref | Ref | Ref | Ref | Ref |
| North | 14,545 (28%) | 2,388 (31%) | 1.20 (1.14-1.27) | 1.19 (1.13-1.26) | 1.17 (1.11-1.24) | 1.07 (1.01-1.13) | 1.12 (1.06-1.19) |
| South East | 15,857 (31%) | 2,404 (32%) | 1.08 (1.03-1.14) | 1.11 (1.06-1.18) | 1.09 (1.03-1.15) | 0.97 (0.92-1.03) | 1.01 (0.96-1.07) |
| **Year of diagnosis** |  |  |  |  |  |  |  |
| 1997-2001 | 10,390 (20%) | 2,705 (36%) | Ref | Ref | Ref | Ref | Ref |
| 2002-2006 | 11,077 (22%) | 2,209 (29%) | 0.84 (0.79-0.89) | 0.86 (0.81-0.91) | 0.86 (0.81-0.91) | 0.79 (0.75-0.84) | 0.79 (0.75-0.84) |
| 2007-2011 | 13,302 (26%) | 1,761 (23%) | 0.69 (0.65-0.73) | 0.73 (0.68-0.77) | 0.72 (0.67-0.76) | 0.68 (0.64-0.72) | 0.68 (0.64-0.73) |
| 2012-2016 | 16,371 (32%) | 917 (12%) | 0.73 (0.67-0.79) | 0.77 (0.71-0.83) | 0.80 (0.74-0.87) | 0.73 (0.67-0.79) | 0.73 (0.67-0.79) |
| **Grade** |  |  |  |  |  |  |  |
| Grade I-(Well) differentiated | 7,465 (14%) | 351 (5%) | Ref |  | Ref | Ref | Ref |
| Grade II- Moderately (well) differentiated | 23,878 (47%) | 2,701 (35%) | 2.78 (2.49-3.11) |  | 1.86 (1.66-2.08) | 1.84 (1.65-2.06) | 1.84 (1.64-2.06) |
| Grade III-Poorly differentiated | 19,797 (39%) | 4,540 (60%) | 6.08 (5.45-6.78) |  | 3.02 (2.70-3.38) | 3.04 (2.71-3.41) | 3.02 (2.70-3.39) |
| **TNM stage** |  |  |  |  |  |  |  |
| I | 20,598 (40%) | 930 (12%) | Ref |  | Ref | Ref | Ref |
| II | 20,722 (40%) | 2,736 (36%) | 2.46 (2.27-2.67) |  | 2.16 (2.00-2.33) | 2.12 (1.96-2.29) | 2.11 (1.95-2.28) |
| III | 7,935 (16%) | 2,801 (37%) | 10.13 (9.21-11.16) |  | 6.33 (5.86-6.84) | 5.95 (5.48-6.45) | 5.90 (5.44-6.40) |
| IV | 1,885 (4%) | 1,125 (15%) | 30.13 (27.61-32.89) |  | 20.94 (19.13-22.91) | 11.05 (9.96-12.25) | 11.09 (10.00-12.30) |
| **Screening** |  |  |  |  |  |  |  |
| Yes | 16,227 (32%) | 853 (11%) | Ref |  | Ref | Ref | Ref |
| No | 34,913 (68%) | 6,739 (89%) | 3.88 (3.62-4.17) |  | 1.76 (1.63-1.90) | 1.63 (1.51-1.76) | 1.62 (1.50-1.75) |

Supplementary Table 1 (continued) Unadjusted and adjusted Cox models for the association of ER status with breast cancer death (page 2 of 2)

|  | **No women (%)** | **No deaths (%)** | **Model 0**  **unadjusted** | **Model 1** | **Model 2** | **Model 3** | **Model 4** |
| --- | --- | --- | --- | --- | --- | --- | --- |
| **Surgery** |  |  |  |  |  |  |  |
| Yes | 48,343 (94%) | 6,204 (82%) | Ref |  |  | Ref | Ref |
| No | 2,797 (6%) | 1,388 (18%) | 10.17 (9.58-10.78) |  |  | 4.04 (3.74-4.37) | 4.01 (3.70-4.33) |
| **Radiotherapy** |  |  |  |  |  |  |  |
| Yes | 35,250 (69%) | 4,863 (64%) | Ref |  |  | Ref |  |
| No | 15,890 (31%) | 2,729 (36%) | 1.25 (1.19-1.31) |  |  | 1.04 (0.99-1.10) | 1.03 (0.98-1.09) |
| **Chemotherapy** |  |  |  |  |  |  |  |
| Yes | 20,329 (40%) | 4,216 (56%) | Ref |  |  | Ref | Ref |
| No | 30,811 (60%) | 3,376 (44%) | 0.55 (0.53-0.58) |  |  | 0.98 (0.92-1.04, p=0.488) | 0.96 (0.90-1.02) |
| **Hormone therapy** |  |  |  |  |  |  |  |
| Yes | 40,136 (79%) | 5,098 (67%) | Ref |  |  | Ref | Ref |
| No | 11,004 (21%) | 2,494 (33%) | 1.97 (1.87-2.06) |  |  | 1.31 (1.21-1.41) | 1.31 (1.21-1.41) |
| **SIMD quintile** |  |  |  |  |  |  |  |
| Least deprived | 11,083 (22%) | 1,481 (19%) | Ref |  |  |  | Ref |
| 4 | 11,121 (22%) | 1,556 (20%) | 1.07 (0.99-1.15) |  |  |  | 1.03 (0.96-1.11) |
| 3 | 10,858 (21%) | 1,643 (22%) | 1.18 (1.10-1.27) |  |  |  | 1.12 (1.04-1.20) |
| 2 | 9,862 (19%) | 1,576 (21%) | 1.28 (1.19-1.37) |  |  |  | 1.19 (1.11-1.28) |
| Most deprived | 8,216 (16%) | 1,336 (18%) | 1.32 (1.23-1.42) |  |  |  | 1.24 (1.15-1.34) |
| **Charlson Score** |  |  |  |  |  |  |  |
| Mean (SD) | 0.0 (0.3) | 0.1 (0.3) | 1.34 (1.24-1.44) |  |  |  | 1.23 (1.13-1.33) |

Footnote: Model 1 was adjusted for age at diagnosis, incidence year and NHS region. Model 2: model 1 + tumour characteristics (grade, TNM stage and method of detection). Model 3= model 2 + treatments (surgery, radiotherapy, chemotherapy and hormone therapy). Model 4= model 3 + SIMD and Charlson score index. Models carried out in the complete case dataset with n=51,140 women, number of BC deaths=7,592. ER= oestrogen receptor, NHS= National Health Service, Ref= reference category, SD= standard deviation, SIMD= Scottish Index of Multiple Deprivation, TNM= tumour, nodes, metastases.

Supplementary Table 2 Traditional unadjusted and adjusted Cox models for the association of IHC defined molecular subtypes with breast cancer death (page 1 of 2)

|  | **No women**  **(%)** | **No deaths (%)** | **Model 0- Unadjusted** | **Model 1** | **Model 2** | **Model 3** | **Model 4** |
| --- | --- | --- | --- | --- | --- | --- | --- |
| **IHC defined subtype** |  |  |  |  |  |  |  |
| Luminal A | 13,755 (56%) | 723 (33%) | Ref | Ref | Ref | Ref | Ref |
| Luminal B | 7,132 (29%) | 753 (35%) | 1.95 (1.76-2.16) | 2.03 (1.83-2.25) | 1.71 (1.54-1.89) | 2.06 (1.84-2.29) | 2.04 (1.83-2.28) |
| HER2-enriched | 1,140 (4%) | 188 (9%) | 3.36 (2.86-3.95) | 3.48 (2.96-4.09) | 2.44 (2.08-2.87) | 1.97 (1.60-2.44) | 1.95 (1.58-2.41) |
| Triple Negative | 2,635 (11%) | 504 (23%) | 4.09 (3.65-4.58) | 4.31 (3.85-4.84) | 4.54 (4.03-5.10) | 4.02 (3.36-4.80) | 3.93 (3.29-4.70) |
| **Age** |  |  |  |  |  |  |  |
| <50 years | 5,037 (20%) | 441 (20%) | 1.47 (1.31-1.66) | 1.26 (1.12-1.42) | 0.87 (0.78-0.99) | 0.94 (0.83-1.06) | 0.94 (0.83-1.07) |
| 50-69 years | 13,332 (54%) | 764 (35%) | Ref | Ref | Ref | Ref | Ref |
| 70 years or older | 6,293 (26%) | 963 (45%) | 3.19 (2.90-3.50) | 3.23 (2.94-3.55) | 2.08 (1.88-2.29) | 1.49 (1.33-1.66) | 1.49 (1.33-1.67) |
| **NHS Scottish region** |  |  |  |  |  |  |  |
| West | 11,072 (45%) | 962 (44%) | Ref | Ref | Ref | Ref | Ref |
| North | 6,699 (27%) | 630 (29%) | 1.04 (0.94-1.15) | 1.07 (0.97-1.19) | 1.15 (1.04-1.27) | 1.19 (1.07-1.32) | 1.26 (1.13-1.40) |
| South East | 6,891 (28%) | 576 (27%) | 0.93 (0.83-1.03) | 0.98 (0.88-1.08) | 0.96 (0.86-1.06) | 1.00 (0.91-1.12) | 1.04 (0.94-1.16) |
| **Year of diagnosis** |  |  |  |  |  |  |  |
| 2009-2011 | 7,425 (30%) | 1,005 (46%) | Ref | Ref | Ref | Ref | Ref |
| 2012-2016 | 17,237 (70%) | 1,163 (54%) | 1.16 (1.06-1.27) | 1.15 (1.05-1.27) | 1.24 (1.13-1.36) | 1.19 (1.08-1.31) | 1.19 (1.08-1.31) |
| **TNM stage** |  |  |  |  |  |  |  |
| I | 10,081 (41%) | 167 (8%) | Ref |  | Ref | Ref | Ref |
| II | 9,824 (40%) | 622 (29%) | 3.92 (3.30-4.65) |  | 2.52 (2.12-3.00) | 2.54 (2.12-3.03) | 2.51 (2.10-3.00) |
| III | 3,323 (13%) | 631 (29%) | 12.33 (10.40-14.62) |  | 7.52 (6.31-8.96) | 7.30 (6.09-8.76) | 7.26 (6.06-8.71) |
| IV | 1,434 (6%) | 748 (34%) | 57.34 (48.46-67.85) |  | 38.12 (32.00-45.41) | 14.54 (11.91-17.76) | 14.72 (12.05-17.97) |
| **Screening** |  |  |  |  |  |  |  |
| Yes | 8,359 (34%) | 157 (7%) | Ref |  | Ref | Ref | Ref |
| No | 16,303 (66%) | 2,011 (93%) | 7.27 (6.18-8.56) |  | 2.58 (2.17-3.06) | 2.16 (1.81-2.58) | 2.13 (1.78-2.53) |

Supplementary Table 2 (continued) Traditional unadjusted and adjusted Cox models for the association of IHC defined molecular subtypes with breast cancer death (page 2 of 2)

|  | **No women**  **(%)** | **No deaths (%)** | **Model 0- Unadjusted** | **Model 1** | **Model 2** | **Model 3** | **Model 4** |
| --- | --- | --- | --- | --- | --- | --- | --- |
| **Surgery** |  |  |  |  |  |  |  |
| Yes | 22,178 (90%) | 1,212 (56%) | Ref |  |  | Ref | Ref |
| No | 2,484 (10%) | 956 (44%) | 13.92 (12.76-15.19) |  |  | 5.06 (4.43-5.76) | 4.98 (4.37-5.68) |
| **Radiotherapy** |  |  |  |  |  |  |  |
| Yes | 17,726 (72%) | 1,173 (54%) | Ref |  |  | Ref |  |
| No | 6,936 (28%) | 995 (46%) | 2.48 (2.27-2.69) |  |  | 1.06 (0.96-1.17) | 1.05 (0.95-1.16) |
| **Chemotherapy** |  |  |  |  |  |  |  |
| Yes | 9,261 (38%) | 1,026 (47%) | Ref |  |  | Ref | Ref |
| No | 15,401 (62%) | 1,142 (53%) | 0.71 (0.65-0.77) |  |  | 1.28 (1.14-1.44) | 1.25 (1.11-1.40) |
| **Hormone therapy** |  |  |  |  |  |  |  |
| Yes | 19,475 (79%) | 1,300 (60%) | Ref |  |  | Ref | Ref |
| No | 5,187 (21%) | 868 (40%) | 2.76 (2.54-3.01) |  |  | 1.84 (1.59-2.14) | 1.85 (1.59-2.14) |
| **SIMD quintile** |  |  |  |  |  |  |  |
| Least deprived | 5,260 (21%) | 399 (18%) | Ref |  |  |  | Ref |
| 4 | 5,277 (21%) | 393 (18%) | 0.98 (0.85-1.13) |  |  |  | 0.96 (0.83-1.10) |
| 3 | 5,126 (21%) | 453 (21%) | 1.19 (1.04-1.37) |  |  |  | 1.11 (0.97-1.27) |
| 2 | 4,784 (20%) | 478 (22%) | 1.39 (1.22-1.59) |  |  |  | 1.16 (1.01-1.32) |
| Most deprived | 4,215 (17%) | 445 (21%) | 1.49 (1.30-1.71) |  |  |  | 1.29 (1.12-1.48) |
| **Charlson Score** |  |  |  |  |  |  |  |
| Mean (SD) | 0.0 (0.3) | 0.1 (0.3) | 1.51 (1.34-1.70) |  |  |  | 1.23 (1.08-1.40) |

Footnote: Model 1 was adjusted for age at diagnosis, incidence year and NHS region. Model 2: model 1 + tumour characteristics (grade, TNM stage and method of detection. Model 3= model 2 + treatments (surgery, radiotherapy, chemotherapy and hormone therapy). Model 4= model 3 + SIMD and Charlson score index. Models carried out in the complete case dataset with n=24,662 women, number of BC deaths=2,168. All HRs were statistically significant at the 0.1% level unless stated otherwise. IHC= immunohistochemistry, NHS= National Health Service, Ref= reference category, SD= standard deviation, SIMD= Scottish Index of Multiple Deprivation, TNM= tumour, nodes, metastases.

Supplementary Table 3 Comparison of fully adjusted Cox model with model with time-varying effects (page 1 of 2)

|  | **Fully adjusted Cox model**  **N=51,140, deaths=7,592** | **Cox model with time by covariate interactions**  **N=51,140, deaths=7,592** | |
| --- | --- | --- | --- |
|  | **HR (95%CI, P value)** | **HR (95%CI, P value)**  **Main effect** | **HR (95%CI, P value)**  **Time-varying effect** |
| **ER Status** |  |  | **ER Status*time** |
| Positive | Ref | Ref | Ref |
| Negative | 1.44 (1.33-1.56) | 2.89 (2.55-3.29) | 0.83 (0.81-0.85) |
| **Age** |  |  |  |
| <50 years | 0.89 (0.84-0.95) | 0.89 (0.84-0.95) |  |
| 50-69 years | Ref | Ref |  |
| 70 years or older | 1.36 (1.27-1.45) | 1.35 (1.27-1.44) |  |
| **NHS region** |  |  |  |
| West | Ref | Ref |  |
| North | 1.12 (1.06-1.19) | 1.11 (1.05-1.17) |  |
| South East | 1.01 (0.96-1.07, p=0.665) | 0.99 (0.94-1.05, p=0.847) |  |
| **Year of diagnosis** |  |  |  |
| 1997-2001 | Ref | Ref |  |
| 2002-2006 | 0.79 (0.75-0.84) | 0.79 (0.74-0.84) |  |
| 2007-2011 | 0.68 (0.64-0.73) | 0.68 (0.64-0.73) |  |
| 2012-2016 | 0.73 (0.67-0.79) | 0.74 (0.68-0.79) |  |
| **Grade** |  |  | **Grade*time** |
| Grade I-(Well) differentiated | Ref | Ref | Ref |
| Grade II- Moderately (well) differentiated | 1.84 (1.64-2.06) | 2.10 (1.72-2.57) | 0.99 (0.96-1.01, p=0.369) |
| Poorly differentiated | 3.02 (2.70-3.39) | 4.96 (4.05-6.08) | 0.91 (0.89-0.94) |

Supplementary Table 3 (continued) Comparison of fully adjusted Cox model with model with time-varying effects (page 2 of 2)

|  | **Fully adjusted Cox model**  **N=51,140, deaths=7,592** | **Cox model with time by covariate interactions**  **N=51,140, deaths=7,592** | |
| --- | --- | --- | --- |
|  | **HR (95%CI, P value)** | **HR (95%CI, P value)**  **Main effect** | **HR (95%CI, P value)**  **Time-varying effect** |
| **TNM stage** |  |  | **TNM stage*time** |
| I | Ref | Ref | Ref |
| II | 2.11 (1.95-2.28) | 2.61 (2.28-2.99) | 0.96 (0.94-0.98) |
| III | 5.90 (5.44-6.40) | 9.13 (7.94-10.50) | 0.91 (0.89-0.93) |
| IV | 11.09 (10.00-12.30) | 23.54 (19.74-28.08) | 0.80 (0.77-0.83) |
| **Screening** |  |  | **Screening*time** |
| Yes | Ref | Ref | Ref |
| No | 1.62 (1.50-1.75) | 2.07 (1.82-2.36) | 0.96 (0.94-0.98) |
| **Surgery** |  |  | **Surgery*time** |
| Yes | Ref | Ref | Ref |
| No | 4.01 (3.70-4.33) | 5.10 (4.49-5.78) | 0.90 (0.87-0.94) |
| **Radiotherapy** |  |  | **Radiotherapy*time** |
| Yes |  |  | Ref |
| No | 1.03 (0.98-1.09, p=0.201) | 1.24 (1.15-1.34) | 0.96 (0.95-0.97) |
| **Chemotherapy** |  |  | **Chemotherapy*time** |
| Yes | Ref | Ref | Ref |
| No | 0.96 (0.90-1.02, p=0.218) | 1.25 (1.14-1.36) | 0.94 (0.93-0.96) |
| **Hormone therapy** |  |  | **Hormone therapy*time** |
| Yes | Ref | Ref | Ref |
| No | 1.31 (1.21-1.41) | 1.69 (1.50-1.92) | 0.93 (0.91-0.96) |
| **SIMD quintile** |  |  |  |
| Least deprived | Ref | Ref |  |
| 4 | 1.03 (0.96-1.11, p=0.371) | 1.03 (0.95-1.10, p=0.371) |  |
| 3 | 1.12 (1.04-1.20, p=0.002) | 1.12 (1.04-1.20, p=0.002) |  |
| 2 | 1.19 (1.11-1.28) | 1.18 (1.10-1.27) |  |
| Most deprived | 1.24 (1.15-1.34) | 1.23 (1.14-1.33) |  |
| **Charlson Score** |  |  |  |
| Mean (SD) | 1.23 (1.13-1.33) | 1.20 (1.11-1.30) |  |

Models include age, incidence year, NHS region, grade, TNM stage, method of detection, surgery, radiotherapy, chemotherapy, hormone therapy, SIMD and Charlson score index. All HRs were statistically significant at the 0.1% level unless stated otherwise. CI= confidence interval, HR= hazard ratio, NHS= National Health Service, SD= standard deviation, SIMD= Scottish Index of Multiple Deprivation, TNM= tumour, nodes, metastases

Supplementary Table 4 Estimates of hazard ratio at 1, 3, 5 and 10 years predicted from Cox model with time-varying covariates

|  | **HR at 1 year** | **HR at 3 years** | **HR at 5 years** | **HR at 10 years** |
| --- | --- | --- | --- | --- |
| **ER Status** |  |  |  |  |
| Positive | Ref | Ref | Ref | Ref |
| Negative | 2.39 (2.08, 2.80) | 1.63 (1.23, 2.03) | 1.12 (0.90, 1.48) | 0.43 (0.31, 0.66) |
| **Grade** |  |  |  |  |
| Grade I-(Well) differentiated | Ref | Ref | Ref | Ref |
| Grade II- Moderately (well) differentiated | 2.08 (1.65, 2.59) | 2.03 (1.52, 2.48) | 1.99 (1.40, 2.69) | 1.90 (1.15, 2.32) |
| Poorly differentiated | 4.53 (3.60, 5.75) | 3.78 (2.83, 5.10) | 3.16 (2.23, 4.53) | 2.01 (1.22, 3.35) |
| **TNM stage** |  |  |  |  |
| I | Ref | Ref | Ref | Ref |
| II | 2.51 (2.27, 2.94) | 2.32 (1.90, 2.83) | 2.14 (1.68, 2.72) | 1.75 (1.25, 2.46) |
| III | 8.33 (7.03, 9.78) | 6.96 (5.53, 8.50) | 5.81 (4.35, 7.39) | 3.71 (2.39, 5.21) |
| IV | 18.92 (15.18, 23.34) | 12.18 (9.03, 15.96) | 7.85 (5.37, 10.91) | 2.61 (1.46, 4.22) |
| **Screening** |  |  |  |  |
| Yes | Ref | Ref | Ref | Ref |
| No | 1.99 (1.72, 2.32) | 1.84 (1.52, 2.23) | 1.70 (1.35, 2.14) | 1.39 (1.01, 1.93) |
| **Surgery** |  |  |  |  |
| Yes | Ref | Ref | Ref | Ref |
| No | 4.57 (3.90, 5.42) | 3.67 (2.94, 4.81) | 2.94 (2.26, 4.26) | 1.70 (1.11, 3.16) |
| **Radiotherapy** |  |  |  |  |
| Yes | Ref | Ref | Ref | Ref |
| No | 1.20 (1.09, 1.30) | 1.11 (0.99, 1.22) | 1.02 (0.90, 1.15) | 0.84 (0.70, 0.99) |
| **Chemotherapy** |  |  |  |  |
| Yes | Ref | Ref | Ref | Ref |
| No | 1.17 (1.06, 1.31) | 1.04 (0.92, 1.21) | 0.92 (0.80, 1.12) | 0.68 (0.57,0.91) |
| **Hormone therapy** |  |  |  |  |
| Yes | Ref | Ref | Ref | Ref |
| No | 1.57 (1.38, 1.84) | 1.36 (1.15, 1.70) | 1.19 (0.96, 1.57) | 0.84 (0.61, 1.28) |

ER= oestrogen receptor, HR= hazard ratio, Ref= reference category, TNM= tumour, nodes, metastases.

The estimated hazard ratio of each covariate as a function of time t is given by: HR(t)=exp(α+βt) where α is the coefficient for the main effect for each specific covariate, β is the coefficient of the interaction of that covariate with time and t represents time in years

Supplementary Table 5 Primary cause of death amongst women diagnosed with breast cancer in Scotland from 1997 to 2016 by age group

| Primary cause of death | <50 years (N=3,213) | 50-69 years (N=8,894) | 70 years or older (N=14,173) | Total (N=26,280) |
| --- | --- | --- | --- | --- |
| Breast cancer | 2,748 (86%) | 5,095 (57%) | 6,139 (43%) | 13,982 (53%) |
| Other cancer | 193 (6%) | 1,449 (16%) | 1,157 (8%) | 2,799 (11%) |
| CVDs | **59 (2%)** | **916 (10%)** | **3,098 (22%)** | 4,073 (16%) |
| Acute Myocardial Infarction | 15 (<1%) | 242 (3%) | 690 (5%) | 947 (4%) |
| Ischaemic Heart Disease | 10 (<1%) | 167 (2%) | 499 (4%) | 676 (3%) |
| Other heart diseases | 19 (<1%) | 243 (3%) | 745 (5%) | 1,007 (4%) |
| Stroke and other | 15 (<1%) | 264 (3%) | 1,164 (8%) | 1,443 (5%) |
| COPD and other respiratory diseases | 42 (1%) | 440 (5%) | 1,141 (8%) | 1,623 (6%) |
| Alzheimer’s/Dementia | 0 (0%) | 134 (2%) | 1,036 (7%) | 1,170 (5%) |
| Mental Health (depression, alcohol disorders, schizophrenia and suicide) | 17 (<1%) | 56 (<1%) | 21 (<1%) | 94 (<1%) |
| Diabetes and other endocrine diseases | <10 (<1%) | <70 (<1%) | 158 (1%) | 230 (<1%) |
| Miscellaneous | **99 (3%)** | **622 (7%)** | **1,232 (9%)** | 1,953 (8%) |
| Accidents | 12 (<1%) | 54 (<1%) | 192 (1%) | 258 (1%) |
| Any other cause of death | 13 (<1%) | 17 (<1%) | 18 (<1%) | 48 (<1%) |
| Benign neoplasm | <10 (<1%) | <20 (<1%) | 46 (<1%) | 70 (<1%) |
| Blood diseases | 0 (0%) | 11 (<1%) | 17 (<1%) | 28 (<1%) |
| Digestive system diseases | 33 (1%) | 270 (3%) | 412 (3%) | 715 (<1%) |
| Infectious disease | 11 (<1%) | 73 (<1%) | 140 (1%) | 224 (<1%) |
| Kidney and genitourinary system diseases | <10 (<1%) | <70 (<1%) | 243 (2%) | 318 (1%) |
| Medical and surgical complications | <10 (<1%) | <10 (<1%) | 10 (<1%) | 21 (<1%) |
| Musculoskeletal system diseases | <10 (<1%) | <30 (<1%) | 57 (<1%) | 81 (<1%) |
| Other endocrine diseases | <10 (<1%) | <10 (<1%) | 36 (<1%) | 50 (<1%) |
| Other nervous system | 13 (<1%) | 88 (1%) | 116 (<1%) | 217 (<1%) |
| Skin diseases | <10 (<1%) | <20 (<1%) | 27 (<1%) | 43 (<1%) |
| Unknown | 40 (1%) | 101 (1%) | 145 (1%) | 286 (1%) |

Values are frequency (% by column) with some cells showing approximate counts to comply with statistical disclosure. CVD= Cardiovascular Disease, COPD= Chronic Obstructive Pulmonary Disease.

Supplementary Figure 1 – DAG of the effect of multiple covariates (age, subtype, stage, screening, treatments and deprivation) in BCSS
